# Young transgenic hMTH1 mice are protected against dietary fat‐induced metabolic stress—implications for enhanced longevity

**DOI:** 10.1111/acel.13605

**Published:** 2022-06-06

**Authors:** Francesca Marcon, Roberta Meschini, Egidio Iorio, Simonetta Palleschi, Gabriele De Luca, Ester Siniscalchi, Luigi Conti, Mattea Chirico, Maria Elena Pisanu, Francesca De Battistis, Barbara Rossi, Anna Minoprio, Alessandro Giuliani, Peter Karran, Margherita Bignami

**Affiliations:** ^1^ 9289 Department of Environment and Health Istituto Superiore di Sanità Rome Italy; ^2^ Department of Ecological and Biological Sciences Tuscia University Viterbo Italy; ^3^ 9289 Core Facilities Istituto Superiore di Sanità Rome Italy; ^4^ 9289 Oncology and Molecular Medicine Istituto Superiore di Sanità Rome Italy; ^5^ Francis Crick Institute London UK

**Keywords:** ageing, Comet assay, DNA damage, life span, micronucleus, metabolic rate, mitochondria, mouse models, oxidative stress

## Abstract

hMTH1 protects against mutation during oxidative stress. It degrades 8‐oxodGTP to exclude potentially mutagenic oxidized guanine from DNA. hMTH1 expression is linked to ageing. Its downregulation in cultured cells accelerates RAS‐induced senescence, and its overexpression in hMTH1‐Tg mice extends lifespan. In this study, we analysed the effects of a brief (5 weeks) high‐fat diet challenge (HFD) in young (2 months old) and adult (7 months old) wild‐type (WT) and hMTH1‐Tg mice. We report that at 2 months, hMTH1 overexpression ameliorated HFD‐induced weight gain, changes in liver metabolism related to mitochondrial dysfunction and oxidative stress. It prevented DNA damage as quantified by a comet assay. At 7 months old, these HFD‐induced effects were less severe and hMTH1‐Tg and WT mice responded similarly. hMTH1 overexpression conferred lifelong protection against micronucleus induction, however. Since the canonical activity of hMTH1 is mutation prevention, we conclude that hMTH1 protects young mice against HFD by reducing genome instability during the early period of rapid growth and maximal gene expression. hMTH1 protection is redundant in the largely non‐growing, differentiated tissues of adult mice. In hMTH1‐Tg mice, expression of a less heavily mutated genome throughout life provides a plausible explanation for their extended longevity.

## Graphical abstract

hMTH1 overexpression countered high fat diet (HFD)‐induced mitochondrial dysfunction. Young hMTH1‐transgenic mice were protected against oxidative stress, DNA and protein damage. hMTH1 overexpression did not affect the response of adult mice to the HFD. hMTH1 overexpression conferred lifelong protection against micronucleus induction. Expression of fewer mutated proteins provides a plausible explanation for hMTH1‐related HFD protection and the extended longevity of hMTH1‐transgenic mice.

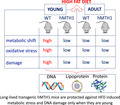

**Abbreviations**
8‐oxoG: 8‐oxo‐7,8‐dihydroguanine
AOPP: advanced oxidation protein product,
BCAA: branched amino acid
BER: base excision repair
DSBs: double‐strand breaks
GPC: glycerophosphocholine
HFD: high‐fat diet
hMTH1: human MTH1
L‐His: L‐histidine
L‐Phe: L‐phenylalanine
L‐Tyr: L‐tyrosine
MDA: malondialdehyde
NHEJ: non‐homologous end joining
PCA: principal component analysis
PCho: phosphocholine
ROS: reactive oxygen species
SD: standard diet
SSBs: single‐strand breaks
WT: wild‐type

## 1 INTRODUCTION

Cellular nucleotide pools are significant targets for the reactive oxygen species (ROS) that are generated during periods of oxidative stress (Haghdoost et al., 2006). To prevent the accumulation of the miscoding base DNA 8‐oxo‐7,8‐dihydroguanine (8‐oxoG—the major guanine oxidation product) during replication, nucleotide hydrolases degrade 8‐oxodGTP to eliminate it as a substrate for DNA polymerases (Ishibashi et al., 2003; Maki & Sekiguchi, 1992; Sakumi et al., 1993). Human MTH1 (hMTH1), the major nucleotide hydrolase activity in human cells, is a homolog of the *E*. *coli* MutT protein, a powerful antimutator (Maki & Sekiguchi, 1992). hMTH1 hydrolyses 8‐oxodGTP (and 2‐oxodATP) to the corresponding monophosphates (Fujikawa et al., 1999). It also acts less efficiently on their ribo‐counterparts to avert the incorporation of oxidized nucleotides into RNA (Hayakawa et al., 1999). ROS also oxidize DNA guanine *in situ*, and base excision repair (BER) initiated at DNA 8‐oxoG:C and A:8‐oxoG pairs by the OGG1 and MUTYH DNA glycosylases, respectively, cooperatively effects the removal of 8‐oxoG (reviewed in Mazzei et al., 2013).

Nucleotide pool sanitization by hMTH1 reduces the incorporation of a potentially mutagenic DNA base. Cells derived from *Mth1* ^−/−^ mice have a twofold to threefold increased spontaneous mutation rate. *Mth1* ^−/−^ mice are cancer‐prone (Tsuzuki et al., 2001) and are particularly susceptible to oxidant‐induced neurotoxicity. The effects of *Mth1* loss on DNA 8‐oxoG levels in mice are subtle, however, and there is no apparent increase in steady‐state DNA 8‐oxoG levels, most likely because of efficient BER (Sakumi et al., 2003). Increased nucleotide hydrolase activity does, however, affect steady‐state DNA 8‐oxoG levels, and in the hMTH1‐Tg mouse that overexpresses hMTH1, these are reduced from an average of around 1,700 to 800 DNA 8‐oxoG per cell in several organs (De Luca et al., 2008, 2013). These modest changes in steady‐state levels of DNA 8‐oxoG are nevertheless associated with significant biological impact, and the hMTH1‐Tg mouse is protected against neurodegeneration in murine models for Parkinson's and Huntington's disease (Yamaguchi et al., 2006; De Luca et al., 2008). Although MTH1 was initially identified as an antimutator that appears to be dispensable for normal cell growth, more recent studies have suggested an essential role in protecting tumour cells from oxidation‐related death (Gad et al., 2014).

Most strikingly, hMTH1 is important in counteracting senescence and ageing. hMTH1 expression is necessary to prevent the rapid onset of cellular senescence associated with forced RAS expression in cultured human cells (Rai et al., 2009). Consistent with these *in vitro* results, hMTH1 overexpressing hMTH1‐Tg mice have a significantly extended lifespan and, most importantly, older transgenic animals retain many behavioural traits of young mice (De Luca et al., 2013).

Since most phenotypes associated with hMTH1 expression are linked to oxidative stress, we used hMTH1‐Tg mice to investigate whether hMTH1 overexpression protects against the consequences of switching to a high‐fat diet (HFD). A HFD is associated with oxidative stress and the induction of lipid and protein oxidation (Rosen & Spiegelman, 2014). We compared the effects of switching to a HFD on growth and on the liver metabolomes of wild‐type (WT) and hMTH1‐Tg mice. The effects of oxidative stress were examined by analysing lipid and protein oxidation, DNA breaks, DNA 8‐oxoG and micronuclei. The response to the HFD was compared in young (2 months old) and adult (7 months old) mice. We report that hMTH1 overexpression alters the metabolic profile in the livers of young mice. It also protects them against the weight gain, metabolic changes and the effects of oxidative stress induced by a HFD challenge. This protection is age‐dependent, however, and is observed only in young (2 months old) animals. By 7 months of age, the effects of HFD challenge in WT adult mice are significantly milder and are similar in both genotypes. hMTH1 overexpression does, however, provide lifelong protection against the age‐dependent increase in micronucleus frequency up to old age (18 months). The surprising selective susceptibility of young animals to the effects of dietary oxidative stress and the age‐dependent protection afforded by hMTH1 overexpression have implications for the mechanism of delayed ageing in the hMTH‐Tg mouse model of extended lifespan.

## 2 RESULTS

### 2.1 Effects of age and genotype on diet‐dependent changes in body weight

**FIGURE 1:**
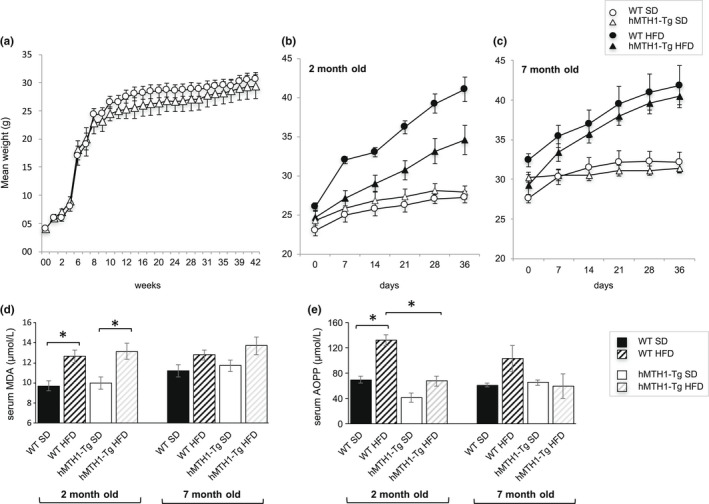
Growth curves and markers of oxidative stress in wild‐type (WT) and hMTH1‐Tg mice. (a) Mean weights of WT (*n* = 5) and hMTH1‐Tg (*n* = 5) animals maintained on a standard diet (SD) were recorded weekly from birth up to 42 weeks. (b) Mean weights of 2‐month‐old animals (*n* = 6 for each experimental condition) during 5 weeks on a SD or high‐fat diet (HFD). Body weights were recorded weekly. (c) As in panel B but showing body weights of 7‐month‐old animals. (d) Serum MDA levels at the end of a 5‐week period on SD or HFD in 2‐ and 7‐month‐old WT and hMTH1‐Tg mice (*n* = 4 per group). (e) As in panel D but showing data for serum AOPP levels. All values are mean ± SE. Asterisk indicates a significant difference (*p* < 0.05, unpaired *t* test)

hMTH1 overexpression did not detectably influence growth rate on the standard diet (SD), and hMTH1‐Tg mice developed normally for the first 10 months of life (Figure 1a). A shift to the HFD at age 2 months induced weight gain in both WT and hMTH1‐Tg mice (Figure 1b). Weight increase was, however, significantly attenuated in the transgenic animals, which over 5 weeks gained around 40% less weight than their wild‐type counterparts (9.7 vs 15 g; *p* = 0.03; interaction diet*genotype: *F* = 16.56; *p* = 0.001). Switching to the HFD at 7 months of age induced a similar weight gain in mice of both genotypes (11.2 and 9.4 g in WT and hMTH1‐Tg, respectively) (*p* = 0.5) (Figure 1c).

We conclude that increased hMTH1 expression provides some protection against HFD‐induced weight gain in young mice. HFD‐challenged adult WT mice gain less weight, and the protection afforded by hMTH1 is largely redundant in adulthood.

### 2.2 Diet and oxidative stress

Oxidative stress was assessed by measuring plasma concentrations of malondialdehyde (MDA) and advanced oxidation protein product (AOPP)‐established biomarkers of lipid and protein oxidation. At 2 months of age on the SD, the steady‐state levels of MDA were comparable in WT and hMTH1‐Tg mice (Figure 1d). The HFD challenge increased MDA levels in both genotypes. The increases were of comparable magnitude (*p* > 0.05). At age 7 months, HFD did not induce a significant increase in MDA in animals of either genotype.

At age 2 months, serum AOPP levels were higher in WT mice than in their hMTH1‐Tg counterparts (*p* = 0.02), possibly indicating a hMTH1‐mediated protection against chronic low‐level oxidative stress (Figure 1e). Consistent with the MDA data, switching to the HFD dramatically increased protein oxidation in WT animals (*p* < 0.001). hMTH1 overexpression protected against this increase, and post‐HFD AOPP levels were significantly lower in hMTH1‐Tg than in WT mice (*p* < 0.05; interaction diet*genotype: *F* = 5.84; *p* = 0.03). In 7‐month‐old mice on the SD, there were no genotype‐dependent differences in either MDA or AOPP levels (Figure 1e). The HFD‐induced MDA and AOPP increases in these adult mice did not reach statistical significance in either genotype.

These measurements confirm that the HFD regime we used induces lipid and protein oxidation. They indicate further that hMTH1 overexpression selectively protects young animals from lipid and protein oxidation. Older animals are less susceptible to HFD‐induced macromolecule oxidation, and hMTH1‐mediated protection is largely redundant in adulthood.

### 2.3 Diet‐dependent changes in liver metabolites

**FIGURE 2:**
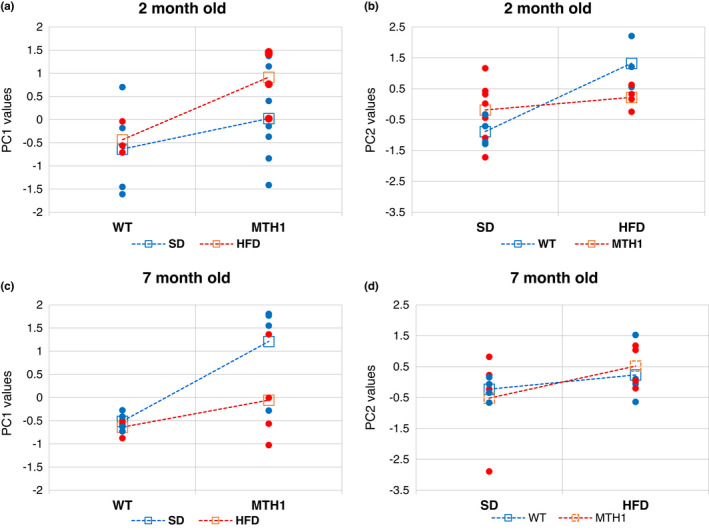
PCA results (score plots). (a) PC1 scores for 2‐month‐old WT and hMTH1‐Tg mice on the standard diet (SD) or a high‐fat diet (HFD). Average values for each genotype are indicated by square symbols. (b) PC2 scores for SD and HFD in 2‐month‐old WT and hMTH1‐Tg mice. Average values for each genotype are indicated by square symbols. (c) PC1 values as in panel A for 7‐month‐old animals. (d) PC2 values as in panel B for 7‐month‐old animals

To examine the changes in metabolic profile induced by a HFD, liver extracts from 2‐ and 7‐month‐old animals of both genotypes were analysed by NMR spectroscopy after a 5‐week HFD challenge. Control animals were maintained on the SD throughout. Twenty‐eight metabolites representing pathways including glucose metabolism, redox balance, amino acid and nucleotide metabolism, and energy production were measured (Tables S1 and S2). Principal component analysis (PCA) in 2‐month‐old SD‐maintained animals revealed significant differences between the genotypes and identified four principal components (PC1‐PC4) that accounted for 83% of the total variance. Their loadings are shown in Table S3. PC1, the most prominent component (47% of explained variance), is an indicator of general metabolic activity (size component). PC1 scores were higher in SD‐ and HFD‐maintained hMTH1‐Tg mice than in their wild‐type counterparts suggesting a more active liver metabolism in the former (genotype: *F* = 5.07; *p* = 0.04) (Figure 2a). Switching to the HFD increased PC1 values in hMTH1‐Tg mice although this did not reach statistical significance (diet: *F* = 1.49; *p* = 0.24; interaction diet*genotype: *F* = 0.6; *p* = 0.45).

PC2 (18% of explained variance) represents the balance between the TCA cycle and lipid metabolism. Positive PC2 values indicate a high phospholipid metabolism and, conversely, a low amino acid metabolism (Table S3). HFD PC2 values are lower in hMTH1‐Tg mice (Figure 2b) indicating a preferential metabolism of amino acids rather than lipids. The shift to HFD significantly increased PC2 values in WT but not in hMTH1‐Tg mice (interaction diet * genotype *F* = 5.8; *p* = 0.03) (Figure 2b). Most of these diet‐related differences were not apparent in adult mice. PCA at 7 months of age (explained variance distribution and component loadings in Table S4) confirmed that PC1 values (general metabolism) were higher in hMTH1‐Tg mice than those in WT animals (genotype: *F* = 9.9; *p* = 0.008) (Figure 2c). The HFD challenge did not significantly affect PC1 and PC2 for adult mice of either genotype (interaction diet*genotype: *F* = 2.4, *p* = 0.15 and *F* = 0.3, *p* = 0.58 for PC1 and PC2, respectively) (Figure 2d).

**FIGURE 3:**
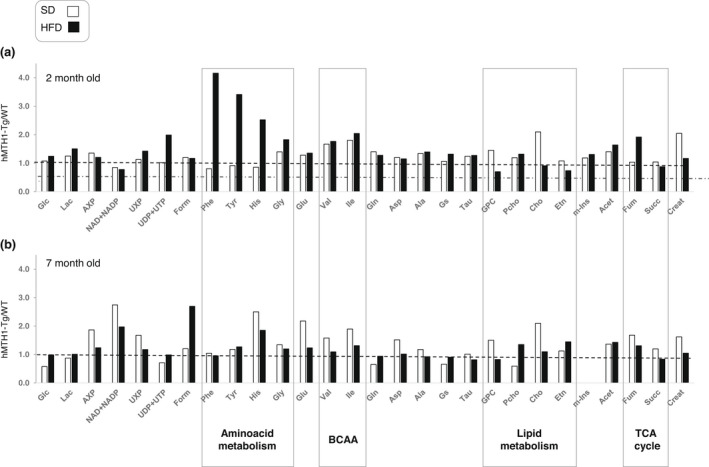
Liver metabolic profiles in WT and hMTH1‐Tg mice as a function of diet and age. (a) The mean values of single metabolites measured in 2‐month‐old animals maintained on the standard diet (SD) or high‐fat diet (HFD) are represented as hMTH1‐Tg:WT ratios. Metabolites belonging to common metabolic pathways are grouped in boxes. (b) As in panel A for 7‐month‐old animals. The absolute quantification of m‐Ins (at 4.01 ppm in proton NMR spectra) was not reported due to the presence of other signals overlapping in the same spectral interval. The indicated metabolites were analysed in the liver of 3–7 animals per group. Abbreviations grouped for metabolic pathway: (a) glucose metabolism: D‐glucose (Glc) and lactic acid (Lac); (b) nucleotide metabolism: adenosine monophosphate (AMP) + adenosine diphosphate (ADP) + adenosine triphosphate (ATP), AXP = (AMP + ADP + ATP), nicotinamide adenine dinucleotide (NAD), nicotinamide adenine dinucleotide phosphate (NADP), uridine monophosphate (UMP) + uridine diphosphate (ADP) + uridine triphosphate (UTP), UXP = (UMP + UDP + UTP); (c) one carbon metabolism: formic acid (Form); (d) amino acid metabolism: L‐phenylalanine (Phe), L‐tyrosine (Tyr), L‐histidine (His), L‐glycine (Gly), L‐glutamic acid (Glu), L‐valine (Val), L‐glutamine (Gln), L‐isoleucine (Ile), L‐Aspartic acid (Asp) and L‐alanine (Ala); (e) redox balance metabolism: glutathione (Gs) and taurine (Tau); (f) lipid metabolism: glycerophosphocholine (GPC), phosphocholine (PCho), free choline (Cho) and ethanolamine (Etn); (g) lipid and amino acid metabolism: acetic acid (Acet); (h) tricarboxylic acid (TCA) cycle: fumaric acid (Fum) and succinic acid (Succ); (i) other pathways: total creatine (creatine + phosphocreatine; Creat)

Metabolite profiles corresponding to SD and HFD are represented as hMTH1‐Tg/WT ratios in Figure 3. At 2 months of age, the levels of branched amino acids (BCAA; L‐valine and L‐isoleucine) and L‐glycine are higher in hMTH1‐Tg mice, particularly on the HFD where the genotype differences reach statistical significance (*p* < 0.05) (Figure 3a and Table S1). BCAAs are known to provide protection against obesity by reducing ROS and stimulating mitochondrial biogenesis (D'Antona et al., 2010). In addition, the major genotype differences induced by HFD involved levels of aromatic amino acids, TCA cycle intermediates and lipid metabolism (Figure 3a). Significantly, all the differences between the two genotypes that we observed at 2 months of age had disappeared by age 7 months (Figure 3b).

**FIGURE 4:**
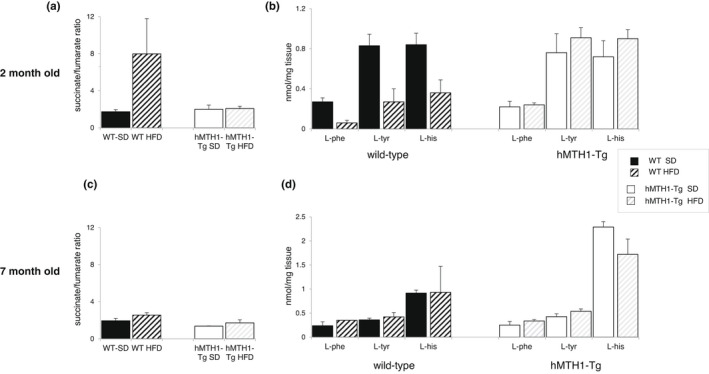
Levels of the most relevant metabolites in WT and hMTH1‐Tg mice as a function of diet and age. (a) Succinate/fumarate ratios in 2‐month‐old animals maintained on the standard diet (SD) or high‐fat diet (HFD). (b) Levels of aromatic amino acids in 2‐month‐old animals maintained on the SD or HFD. (c) As in panel A for 7‐month‐old animals. (d) As in panel B for 7‐month‐old animals. The indicated metabolites were analysed in the liver of 3–7 animals per group. L‐Phe: L‐phenylalanine; L‐Tyr: L‐tyrosine; and L‐His, L‐histidine. All values are mean ± SE

In young wild‐type mice, the HFD increased the succinate/fumarate ratio from 2 to 8, whereas this ratio remained unchanged in hMTH1‐Tg mice (1.8 and 1.9) (interaction diet*genotype: *F* = 5.22; *p* = 0.04) (Figure 4a). In addition, a HFD‐induced reduction in the levels of the fumarate precursors L‐phenylalanine (L‐Phe) and L‐tyrosine (L‐Tyr) and L‐histidine (L‐His), a precursor of alpha‐ketoglutarate, occurred only in WT mice (interaction diet^*^genotype: *F* = 7.20; *p* = 0.02 for L‐Phe; *F* = 5.53; *p* = 0.03 for L‐Tyr; and *F* = 5.91; *p* = 0.03 for L‐His) (Figure 4b). Taken together with the PCA, these findings are consistent with a HFD‐induced impairment of the TCA cycle in young WT mice against which hMTH1 overexpression provides protection. Since inhibition of TCA cycle enzymes—and particularly an increase in succinate/fumarate ratio—is associated with mitochondrial damage (An et al., 2013), these data are consistent with hMTH1 overexpression providing protection against HFD‐induced mitochondrial dysfunction. Importantly, none of the differences in the metabolic profiles of the two genotypes at 2 months of age were observed in 7‐month‐old mice (Figure 4c,d).

As indicated by PCA and by the quantitative analyses of metabolites, the HFD also differently affected the phospholipid turnover in the two genotypes at 2 months of age. The average doubling of glycerophosphocholine (GPC) content in WT mice (0.8 vs 1.6 on the SD and HFD, respectively; Table S1) suggests the activation of phospholipid catabolism associated with the induction of stress signalling pathways (Farber et al., 2000). This increase does not occur in hMTH1‐Tg mice (1.12 and 1.11 on the SD and HFD). In contrast, phosphocholine (PCho) levels almost doubled (0.98 vs 1.73 on the SD and HFD) in hMTH1‐Tg mice indicating the activation of phosphatidylcholine synthesis, a major component of phospholipid membranes.

In summary, hMTH1 overexpression is associated with a generally more active metabolism in young mouse liver. It also prevents HFD‐induced excess weight gain, TCA cycle impairment and changes in phospholipid metabolism that are associated with stress signalling. These genotype‐dependent differences were confined to young mice and were not observed in adult animals. In terms of liver metabolism, hMTH1 overexpression appears to be advantageous at 2 months of age and this advantage is magnified under a HFD challenge. By adulthood, the advantage from hMTH1 overexpression has become redundant.

### 2.4 Fasting‐induced changes in liver metabolites and markers of oxidative stress

**FIGURE 5:**
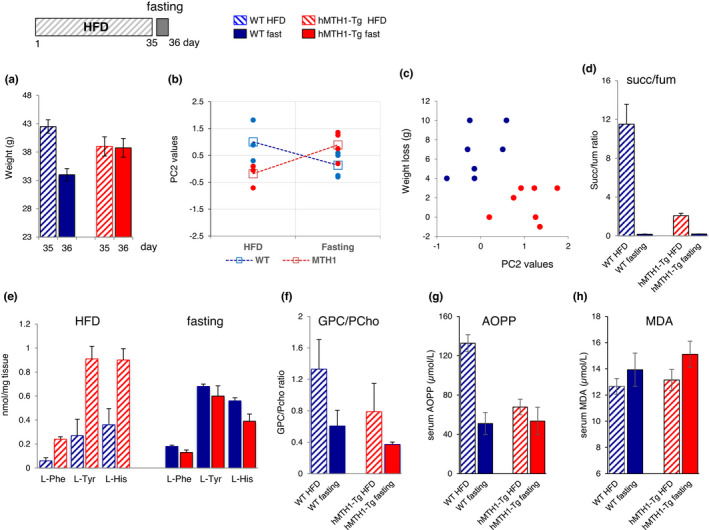
Effects of a brief fasting period on body weight, metabolic profiles and markers of oxidative stress in 2‐month‐old animals. Scheme of the fasting experiment is shown on the top of the figure. After 35 days in high‐fat diet (HFD), mice were fasted for further 20 h. Animals were sacrificed on Day 36. (a) Mean body weights were recorded at days 35 (end of HFD) and 36 (end of fasting). (b) Values of PC2 scores for HFD and fasting in WT mice and hMTH1‐Tg mice. Average values for each genotype are indicated by square symbols. (c) Discrimination of WT and hMTH1‐Tg mice by weight loss and PC2 scores. (d‐e‐f‐g‐h) Comparison of metabolite levels in HFD and fasted WT and hMTH1‐Tg mice. (d) Succinate/fumarate ratios. (e) L‐Phe, L‐Tyr and L‐His levels. (f) Comparison of GPC/PCho ratios. (g) Comparison of AOPP levels. (h) Comparison of MDA levels. Mean values of 4 animals/group are reported ± SE. Asterisks indicate significant changes (**p < 0.01, unpaired *t* test)

The above liver metabolomic findings indicate that the different susceptibilities to HFD challenge of young WT and hMTH1‐Tg mice were mostly erased by 5 months of additional growth on the SD. Brief post‐HFD fasting had a similar effect in young mice. Thus, a 20‐h fast following a HFD challenge induced a dramatic (20%) decrease in body mass (from 42.5 to 34 g) (*p* = 0.002) in 2‐month‐old WT mice (Figure 5a). Fasting did not measurably affect the weight of HFD‐challenged hMTH1‐Tg mice (Figure 5a), and the weight loss in control SD‐maintained mice was much less marked (4.3 and 2 g in WT and hMH1‐Tg mice, respectively).

PCA of post‐fasting HFD liver metabolite patterns (Table S5) identified five principal components accounting for 79% of total variance (Table S6). PC1 scores (40% of explained variance) were higher in fasted hMTH1‐Tg mice (genotype effect: *F* = 6.64; *p* = 0.01) confirming the more active liver metabolism of this group. PC2 (16% of explained variance—accounting for the balance between TCA cycle and phospholipid metabolism) had comparable scores in WT and hMTH‐Tg mice reflecting a fasting‐induced decrease in WT and an increase in hMTH1‐Tg values (Figure 5b). The clear separation between the two genotypes is evident in the scatter plot of PC2 vs weight loss (Pearson's coefficient −0.58, *p* = 0.029) (Figure 5c).

Negative PC2 values are associated with high level of weight loss in WT mice. In contrast, positive PC2 values are related to minimal weight loss in hMTH‐Tg mice (Figure 5c). Fasting reversed the HFD‐mediated impairment of the TCA cycle in WT mice. It returned the average succinate/fumarate ratio to a normal value (8 vs 0.17 pre‐ and post‐fasting, respectively, Figure 5d). Consistent with these restored TCA enzyme activities, the levels of L‐Phe, L‐Tyr and L‐His increased and were comparable to those of hMTH1‐Tg mice (Figure 5e). Fasting also reset the WT GPC/PCho ratio (1.3 vs 0.6 pre‐ and post‐fasting, respectively) consistent with fasting deactivating the HFD‐induced stress signalling (Figure 5f). Finally, post‐HFD fasting completely reversed diet‐induced protein oxidation in WT animals, although no effect on diet‐induced lipid oxidation was apparent (Figure 5g,h). As expected, fasting had no detectable effect on AOPP levels in hMTH1‐Tg mice. In summary, fasting reverses the mitochondrial dysfunction induced by HFD against which hMTH1 overexpression provides protection.

### 2.5 Age‐ and diet‐dependent changes in DNA damage

The alkaline comet assay was used to measure levels of DNA damage in peripheral blood lymphocytes as a function of age, diet and genotype.

**FIGURE 6:**
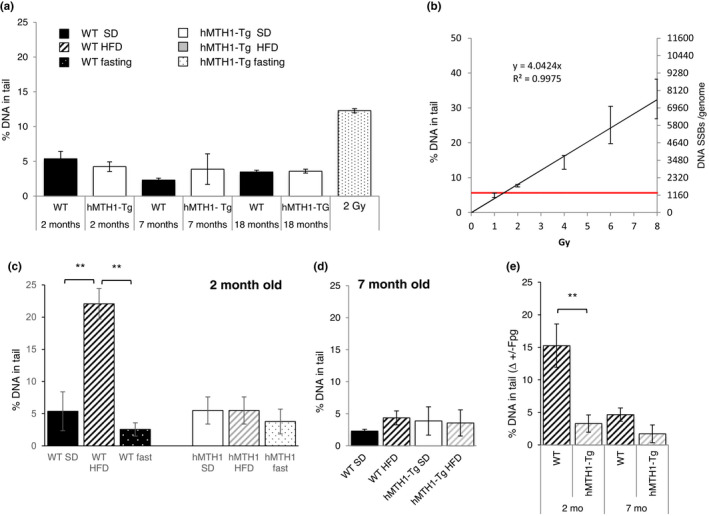
Analysis of DNA damage by alkaline comet assays on peripheral blood cells of WT and hMTH1‐Tg mice as a function of age and diet. (a) Percentage of tail DNA in blood cells from 2‐month‐, 7‐month‐ and 18‐month‐old WT and hMTH1‐Tg mice maintained on the standard diet (SD). Cells from WT mice (*n* = 3) exposed to 2 Gy of 137Cs γ radiation were used as a positive control. (b) Calibration curve relating the percentage of tail DNA to single‐strand breaks (SSBs) per genome. A dose–response curve was obtained by treatment of blood cells with ionizing radiation. The background levels of the percentage of tail DNA revealed by the comet assay (red line) correspond to the damage induced by 1.1 Gy gamma‐rays or around 1300 DNA SSBs per genome (c) Percentage of tail DNA in blood cells from 2‐month‐old WT and hMTH1‐Tg mice maintained on the SD, high‐fat diet (HFD) and 20‐h fasting following a 5‐week HFD. (d) Percentage of tail DNA in blood cells from 7‐month‐old WT and hMTH1‐Tg mice on the SD or HFD (e) DNA 8‐oxodG measured by the comet assay in the presence of the Fpg DNA glycosylase/AP endonuclease. DNA 8‐oxodG levels are calculated as the increase in the percentage of tail DNA induced by Fpg over background level in 2‐month‐ and 7‐month‐old WT and hMTH1‐Tg mice. All values are mean ± SE. The comet assay parameters in different experimental groups were compared by two‐tailed Student's *t* test. Asterisks indicate significant differences (**p < 0.01)

The spontaneous levels of DNA damage were not significantly affected by ageing and were similar in young, adult and old mice (Figure 6a). In particular, the extent of DNA breakage in the two genotypes was comparable at 2 and 7 months of age (% tail DNA in the ranges 2.3–5.4 and 3.9–4.2 in WT and hMTH1‐Tg mice, respectively). The same analysis in old animals (18 months of age) revealed a similarly low number of single‐strand breaks (SSBs), indicating that the steady‐state level of lymphocyte DNA SSBs remains low into old age and is not detectably influenced by hMTH1 status. These values were close to the limit of detection of the comet assay at around 1300 DNA SSBs per genome as calculated from the calibration curve (Figure 6b).

A HFD challenge at age 2 months increased DNA breakage approximately fourfold in WT mice leading to around 6000 SSBs per genome (*p* < 0.01; interaction diet*genotype: *F* = 9.61; *p* = 0.004) (Figure 6c). hMTH1 overexpression provided complete protection against this damage as SSBs in HFD‐challenged 2‐month‐old hMTH1‐Tg mice remained at background levels (Figure 6c). In contrast, HFD challenge at 7 months of age did not induce an increase in DNA strand breaks in either genotype (Figure 6d).

A 20‐h fast reduced the high levels of SSBs in young HFD‐challenged WT animals to background values. Fasting did not change the low level of SSBs detected in HFD‐challenged hMTH1‐Tg mice (Figure 6c).

The presence of the major DNA oxidation product, DNA 8‐oxoG, was addressed by the modified comet assays using the Fpg DNA glycosylase/AP endonuclease to reveal the presence of 8‐oxoG:C base pairs. Following HFD challenge, the modified comet assay revealed a statistically significant increase in tail DNA in 2‐month‐old WT mice (*p* = 0.002; interaction diet*genotype: *F* = 8.34; *p* = 0.01) (Figure 6e), highlighting the presence of HFD‐induced 8‐oxoG:C base pairs. This increase corresponds to >4000 SSBs per genome, based on the calibration curve (Figure 6b).

Conversely, in HFD‐challenged hMTH1‐Tg mice of the same age, there was no detectable increase over the background level of Fpg‐sensitive sites, indicating that hMTH1 overexpression protected against HFD‐induced oxidative DNA damage (Figure 6e). In adult (7 months old) mice of both genotypes, there was no HFD‐mediated increase in Fpg‐dependent DNA SSBs, indicating levels of DNA 8‐oxoG close to background values (Figure 6e).

On the SD, at 2 months of age, the level of Fpg‐sensitive sites in blood cells was lower in hMTH1‐Tg mice than in their wild‐type counterparts (1.9 vs 3.0 in hMTH1‐Tg and wild‐type, respectively; *p* = 0.003). These findings confirm previously reported HPLC/EC measurements of DNA 8‐oxodG in hMTH1‐Tg animals (De Luca et al., 2008, 2013). They are consistent with the somewhat higher level of oxidative stress in SD‐maintained young WT animals inferred from their higher serum AOPP levels (Figure 1e). The modified comet assay did not reveal genotype‐dependent differences in Fpg‐sensitive sites in blood cells from SD‐maintained mice at 7 months of age.

These data indicate that in young mice, hMTH1 overexpression allows SD‐maintained mice to avoid a low‐level oxidative stress. It also prevents the induction of SSBs and DNA 8‐oxoG by HFD‐induced oxidative stress. In contrast, adult WT mice do not accumulate more HFD‐induced oxidative DNA damage and are indistinguishable from hMTH1‐Tg mice in this regard. A 20‐h oxidative stress‐free fast is sufficient to allow HFD‐challenged young WT mice to completely reverse their accumulated oxidation‐induced DNA damage.

### 2.6 Age‐ and diet‐dependent changes in micronucleus induction

**FIGURE 7:**
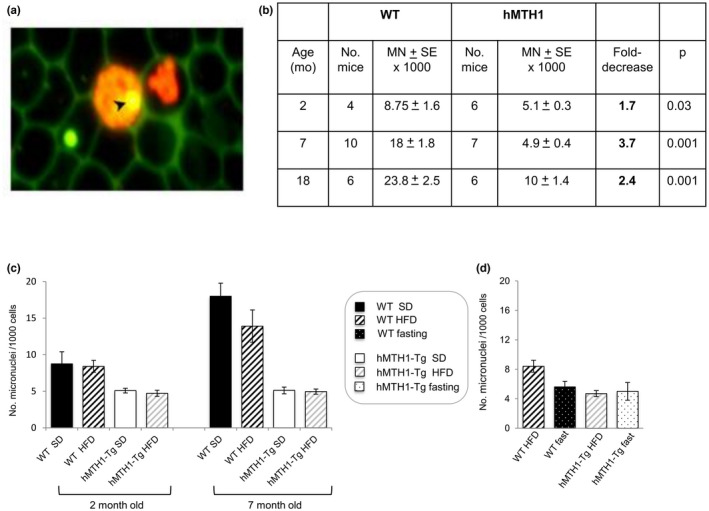
Analysis of micronucleus frequency in peripheral blood reticulocytes of WT and hMTH1‐Tg mice as a function of age and diet. (a) Peripheral blood cells were stained with acridine orange allowing the simultaneous detection of RNA (stained red) and DNA (stained yellowish green). In the picture, two immature reticulocytes can be identified by the red‐fluorescing reticulum structure (red cells); one reticulocyte contains a yellowish green‐fluorescing micronucleus (arrow). Erythrocytes are seen in the background as green‐bordered unstained black cells. A green‐fluorescing micronucleus can also be observed in one erythrocyte. (b) Frequency of micronucleated reticulocytes in 2‐month‐, 7‐month‐ and 18‐month‐old mice maintained on the standard diet (SD). (c) Frequency of micronucleated reticulocytes in WT and hMTH1‐Tg mice maintained on the SD and high‐fat diet (HFD). (d) Effects of fasting on the frequency of micronucleated reticulocytes. Micronuclei were analysed in 2‐month‐old WT and hMTH1‐Tg mice before and after an overnight fast (20 h) following a 5‐week HFD. The experimental design is represented in Figure 5. All values are mean ± SE. Different experimental groups were compared by two‐tailed Student's *t* test

To examine the effects of hMTH1 overexpression on chromosomal damage, we measured the frequency of micronuclei in peripheral blood reticulocytes (an example is shown in Figure 7a) from SD‐maintained mice of two, seven and 18 months of age. The frequency of micronuclei in WT mice increased progressively with age. It doubled between ages 2 and 7 months and was increased by around threefold at 18 months of age. hMTH1 overexpression significantly reduced micronucleus frequency at all ages (interaction age*genotype: *F* = 6.39; *p* = 0.003) (Figure 7b).

Strikingly, micronucleus induction was not detectably affected by a shift to HFD at either 2 or 7 months of age (Figure 7c). Post‐HFD fasting (20 h) at 2 months old reduced the micronucleus frequency in WT animals to values comparable to those in hMTH1‐Tg mice. No changes in the levels of micronuclei were observed in fasted hMTH1‐Tg mice (Figure 7d).

To examine whether micronuclei originated from chromosome loss or chromosome breakage, slides were probed with a fluorescent anti‐kinetochore antibody (CREST). To maximize micronucleus numbers, we analysed blood from 18‐month‐old mice. The frequencies of micronuclei due to chromosome loss (kinetochore‐positive) and those produced by chromosome breakage (kinetochore negative) did not differ significantly between WT and hMTH1‐Tg mice (10% and 11% kinetochore‐positive in WT and hMTH1, respectively) indicating that hMTH1 overexpression protects against the formation of micronuclei by both chromosome breakage and protein damage.

We conclude that hMTH1 overexpression significantly protects against age‐associated micronucleus formation. Micronucleus induction is not, however, influenced by the DNA damage induced by acute oxidative stress associated with the HFD. We note, however, that even though micronucleus induction was clearly independent of changes induced by the HFD, fasting reduced the micronucleus frequency in young WT mice to a value comparable to that in hMTH1‐Tg mice.

It appears therefore that fasting may also reverse the non‐HFD‐dependent damage to macromolecules that affects chromosome stability.

## 3 DISCUSSION

Our findings reveal a surprising connection between nucleotide pool editing and metabolism. The principal component analysis of NMR‐determined metabolite levels demonstrated an association between hMTH1 overexpression, a metabolic reprogramming and more active overall liver metabolism in young mice maintained on the SD. hMTH1 overexpression was particularly advantageous to HFD‐challenged young mice. It prevented the metabolic changes, systemic oxidative stress and excessive weight gain generally observed in obese mice (Manna & Jain, 2015). hMTH1‐mediated protection was not apparent in adult mice in which the HFD challenge induced less dramatic metabolic changes that were not associated with either excessive weight gain or significant oxidative stress.

HFD‐induced oxidative stress in young WT mice was accompanied by an increased liver succinate:fumarate ratio consistent with succinate dehydrogenase inhibition and an impaired TCA cycle (An et al., 2013). hMTH1‐Tg mice are also protected against 3‐nitropropionic acid, a mitochondrial toxin and succinate dehydrogenase inhibitor that induces oxidative DNA damage (De Luca et al., 2008). Mitochondrial dysfunction is a characteristic outcome of a chronic HFD (De Mello et al., 2018), and our findings indicate that a relatively brief (5‐week) HFD challenge also induces mitochondrial dysfunction in young WT but not hMTH1‐Tg mice. The protection conferred by hMTH1 overexpression against mitochondrial insult is consistent with previously reported hMTH1‐mediated prevention of a huntingtin‐related decrease in mitochondrial membrane potential, alterations in mitochondrial morphology and delayed increase in mitochondrial ROS in a cellular model for Huntington's disease (Ventura et al., 2013). Enhanced protection against oxidative mitochondrial DNA damage by mitochondrially targeted overexpressed OGG1 induces a similarly favourable metabolic phenotype with improved mitochondrial energetics and a reduced body weight in a mouse model of obesity (Komakula et al., 2018). Metabolic rewiring occurs also following mutations in genes involved in the DNA damage response and DNA repair (Pascucci et al., 2012).

HFD‐induced mitochondrial dysfunction in young WT animals was accompanied by systemic oxidative stress and increased MDA and AOPP levels. hMTH1 overexpression protected against HFD‐induced protein oxidation, while protection was not evident from MDA measurements. A similar dissociated response was also induced by fasting. The reason for the apparent discrepancy between MDA and AOPP results might lie in the different nature of the two markers. MDA is formed as a result of direct oxidative attack to unsaturated plasma lipids, and its levels are affected by both the quality and the quantity of circulating lipids, the levels of which are directly affected by fat intake *via* the HFD (Tsikas, 2017). Conversely, AOPPs are only indirectly affected by HFD and result from the oxidation of plasma proteins mainly due to inflammation and immune deregulation (Cristani et al., 2016). How hMTH1 differentially modulates MDA and AOPP levels following HFD exposure to young animals is currently unknown. It could be hypothesized that hMTH1, though not affecting plasma lipid peroxidation, might reduce AOPP formation limiting the cascade of pro‐oxidant events triggered by HFD (e.g., inflammatory response). Notwithstanding the discrepancy between MDA and AOPP results, both assays clearly indicated that adult animals do not suffer the HFD‐induced oxidation of serum lipids and proteins that occurs in young mice.

In addition to protein and lipid oxidation, HFD‐induced oxidative stress caused a large increase in DNA strand breaks and DNA 8‐oxoG in peripheral blood cells of young WT mice. In the HFD‐challenged hMTH1‐Tg mice and in adult animals of both genotypes, SSBs and DNA 8‐oxoG levels remained at background values.

**FIGURE 8:**
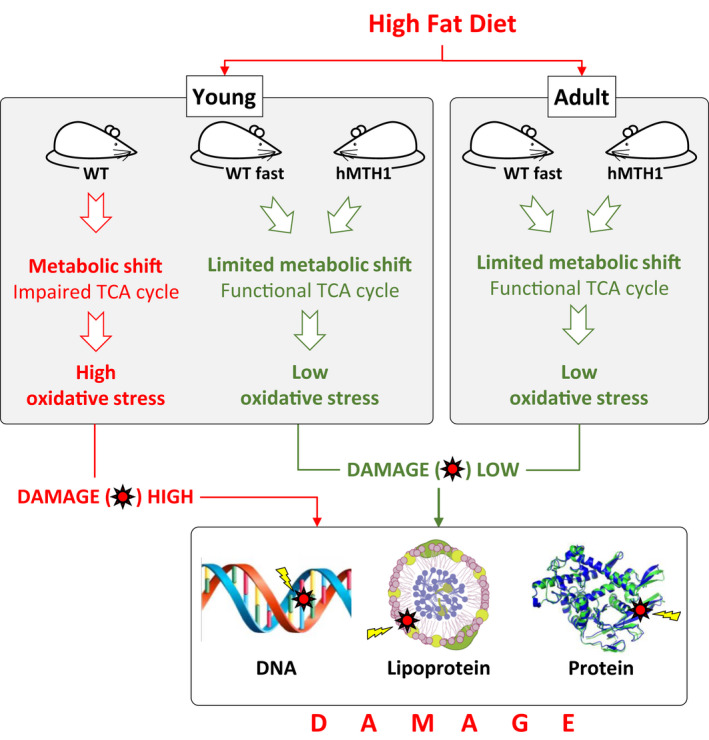
Graphical scheme. Long‐lived transgenic hMTH1 mice are protected against dietary fat‐induced metabolic stress and DNA damage only when they are young

The effects of the HFD in young WT mice were largely reversible, and a brief period of fasting reset their liver metabolite profile more closely to pre‐HFD levels. Other fasting‐related changes included normalization of the succinate: fumarate ratio indicating restoration of a normal TCA cycle. Fasting also induced significant weight loss, consistent with the catabolism of accumulated cytoplasmic lipids. The disappearance of oxidized serum proteins and lymphocyte DNA damage in fasted mice was consistent with the absence of ongoing oxidative stress that allowed time for their respective degradation and repair. Fasting had little impact in hMTH1‐Tg mice in which there was no evidence of HFD‐induced DNA damage or protein oxidation. Thus, the changes induced by a brief fasting period in young HFD‐treated WT mice effectively recapitulate the protection provided by hMTH1 overexpression (Figure 8).

One surprising finding was that the HFD‐induced SSBs in young WT mice did not result in an increase in micronucleus frequency. We infer that HFD‐induced oxidative stress does not promote the formation of sufficient DSBs to increase micronucleus frequency over the background level and that HFD‐induced DNA strand breaks are mainly repairable SSBs. This conclusion is supported by several studies showing that although there is an association between DNA SSBs and HFD‐related body weight increase, no equivalent clear association exists for DSBs (Setayesh et al., 2018). In addition, age‐dependent micronucleus accumulation is not reflected in a measurable age‐dependent increase in steady‐state DNA SSBs (Azqueta et al., 2020). The increased frequency of micronuclei with age is well established (Bonassi et al., 2001), and the significantly reduced rate of micronucleus accumulation that we observe in hMTH1‐Tg mice is consistent with their extended healthy lifespan (de Luca et al., 2013). Micronuclei can reflect chromosome breakage triggered by DNA double‐strand breaks (DSBs) (Fenech et al., 2011), and DSB repair by homologous recombination and non‐homologous end joining (NHEJ) declines with age in several tissues (Garm et al., 2013). Recent findings indicate that ribonucleotide incorporation into DNA (Pryor et al., 2018) and/or damage‐induced long non‐coding RNA (Michelini et al., 2017) promotes the successful processing of DSBs. In addition, oxidized ribonucleotides (8‐oxo‐rGTP) can be efficiently incorporated by DNA polymerase μ during DSB repair by NHEJ (Jamsen et al., 2021) resulting in substrates containing lesions in both DNA strands. Improved destruction of oxidized ribonucleoside triphosphates by overexpressed hMTH1 might reduce the incorporation of potentially inhibitory oxidized ribonucleotides, thereby promoting efficient DSB rejoining and preventing micronucleus formation. This possibility remains to be experimentally tested.

Micronuclei can also result from chromosome loss. It is generally accepted that damage to proteins of the mitotic apparatus (microtubules, kinetochore proteins and spindle checkpoints) can promote chromosome missegregation and micronucleus formation (Fenech et al., 2011). Our analysis of kinetochore‐positive micronuclei (associated with the loss of whole chromosomes) revealed a similar frequency in old WT and hMTH1‐Tg mice. This finding is consistent with hMTH1 overexpression protecting against micronucleus formation by both chromosome breakage events and by mitotic protein damage that promotes chromosome missegregation. This observation provides the first indication that hMTH1 overexpression helps prevent the age‐dependent accumulation of deleterious damaged proteins.

The different responses of young and adult mice to the HFD challenge emphasize the importance of early events in mouse development during which both replication and transcription are at their most active. Our findings demonstrate that improved nucleotide pool editing by overexpressed hMTH1 is selectively protective in young mice. By degrading 8‐oxoGTP, hMTH1 can prevent the incorporation of 8‐oxoG into mRNA during transcription. The presence of 8‐oxoG in mRNA can induce translation errors resulting in the production of altered proteins prone to misfolding (Taddei et al., 1997), although translation rates are severely compromised (Simms et al., 2014). Transcriptional miscoding by DNA 8‐oxodG is another potential source of aberrant mRNA and mutated proteins (Dai et al., 2018). In this case, translation rates would remain unaffected. Although they can potentially alter phenotype, neither misincorporation into mRNA nor 8‐oxoG‐mediated transcriptional mutagenesis are mutagenic in a strict sense. On the contrary, the canonical activity of hMTH1 in degrading 8‐oxodGTP (and 2‐oxodATP) directly impacts DNA replication and mutation. The enhanced antimutagenicity of overexpressed hMTH1 will be maximal during the rapid growth period of approximately 11 weeks post‐conception during which mice attain 75%–80% of their adult body weight and acquire around two thirds of lifetime mutations (Paashuis‐Lew & Heddle, 1998). We suggest that by expressing an increasingly mutated genome at demanding levels, young mice suffer stress related to the production of mutated, misfolded proteins during the rapid growth phase. This renders them particularly susceptible to the effects of a HFD challenge. With fewer mutations, the burden of misfolded proteins expressed by hMTH1‐Tg mice is lower. Misfolded proteins are susceptible to harmful oxidation and aggregation (Tanase et al., 2016), and protein oxidation has been linked to ageing and to age‐related pathologies (Krisko & Radman, 2019). The impact of hMTH1 expression will be considerably less in the largely postmitotic and differentiated tissues of adult mice—consistent with the different responses of young and adult mice to the HFD. Adult WT mice express fewer mutated proteins, and the advantage from hMTH1 overexpression is nullified.

A reduced burden of mutations acquired in early life provides a plausible explanation for the most dramatic phenotype of hMTH1‐Tg mice, their extended lifespan. Events in the first few weeks *post partum* are firmly linked to mouse longevity. Truncating this key growth period, either by genetically altering growth hormone function (reviewed in Bartke & Brown‐Borg, 2004) or by caloric restriction (Sohal & Forster, 2014), extends lifespan and causes dwarfing. In contrast, lifespan extension in hMTH1‐Tg mice is associated with an early growth phase of normal duration and the attainment of full adult size. We suggest that the lifespan of dwarf mice is extended because they acquire fewer mutations owing to a truncated early rapid growth period. The rapid growth period in the hMTH1‐Tg mice is of normal duration, but fewer mutations accumulate because of the antimutagenic effect of enhanced nucleotide pool editing. An extended lifespan is therefore compatible with a normal adult size only in these “antimutator” mice. In this model, ageing at least partly reflects the impact of inescapable later‐life events on proteins encoded by a genome bearing mutations acquired *in utero* and during the first few weeks after birth.

## 4 MATERIALS AND METHODS

### 4.1 Animal care and genotyping

All animal procedures were carried out according to EU Directive 86/609/EEC and to Italian legislation on animal experimentation. The previously described hMTH1‐Tg mice (De Luca et al., 2008) are in a C57BL/6J background. All animals were housed under standardized temperature, humidity and on a 12‐h light‐to‐12‐h dark cycle from 7:00 am to 7:00 pm (light) and 7:00 pm to 7:00 am (dark) with free access to water and food. For diet studies, groups (wild‐type and hMTH1‐Tg) of 2‐month‐ and 7‐month‐old male mice were given ad libitum access to a standard diet (3876 kCal/kg) (Mucedola, Italy) or a high‐fat diet (5,100 kCal/kg) (Mucedola, Italy; Envigo, Madison, WI; MD.06414 Adjusted Calories Diet (60% fat); 60% fat, 20% protein, 20% carbohydrate) for 5 weeks.

### 4.2 Oxidative stress biomarkers

AOPP and total (free and protein‐bound) MDA were determined in serum. Freshly prepared serum was distributed in aliquots and stored at −80°C until analysis. Serum AOPP levels were measured by a photometric procedure (Hanasand et al., 2012) adapted to work with serum volumes as low as 20 µl by using micro UV‐cuvette (BRAND^®^). Chloramine‐T standard (0–180 µM) and potassium iodide solutions were prepared fresh daily. AOPP concentrations were calculated by using a linear regression model and expressed as μM chloramine‐T equivalents. Inter‐assay CV value was 3%.

MDA determination was carried out by RP‐HPLC‐UV analysis according to Moselhy et al. (2013) with minor modifications. The HPLC system consisted of a quaternary pump unit with autosampler, column oven and a diode‐array detector (Flexar, Perkin Elmer). Briefly, 5 µl of 6.6 M NaOH was added to 50 µl serum and incubated at 60°C for 30 min (alkaline hydrolysis step). Samples were then transferred to ice and mixed with 400 µl of 0.22 M sulphuric acid and 150 µl of 200 g/L trichloroacetic acid solution. After 5 min, samples were centrifuged (13,000 *g*, 5 min, 4°C) and 400 µl of each supernatant was transferred to a clean tube, mixed with 200 µl of 0.35% (w/v) thiobarbituric acid (TBA) in NaOH 0.2 M and incubated for 40 min at 90°C (derivatization step). Samples were then cooled and transferred in autosampler vials for HPLC analysis. MDA‐TBA adduct separation was carried out by injecting 10 µl of sample into a ODS‐2 column (Spherisorb, 150 × 4.6 mm, 5 μm particle size; Supelco), protected by a guard cartridge (7.5 × 4.6 mm, Adsorbosphere C18 5 µm, Alltech). The mobile phase consisted of 0.05 M acetate buffer (pH 4.8) containing 20% acetonitrile. Chromatographic runs were carried out isocratically (0.9 ml/min) at constant temperature (28°C), and the MDA‐TBA adduct was detected by absorbance at 532 nm. 1,1,3,3‐tetraethoxypropane solutions in water were used as external standard (2–25 µM), and sample concentration was calculated from peak areas by a linear calibration model. Inter‐assay CV value was 5%.

In both AOPP and MDA assay, run‐to‐run reproducibility was ensured by testing at least two independent internal quality control samples in each run.

### 4.3 Organ sampling and preparation of biological samples

At the end of the 5‐week diet regimen, mice were anaesthetized and blood (~1.0 ml) was taken by cardiac puncture for the preparation of blood smears for the analysis of micronuclei and the isolation of serum for biochemical analysis. The animals were then sacrificed by cervical dislocation, and livers were aseptically removed. Organs were immediately frozen in liquid nitrogen and stored at −80°C for subsequent analyses.

### 4.4 NMR tissue metabolomics

Liver samples were frozen in liquid nitrogen. Lipid and polar metabolites were extracted using the dual‐phase extraction method. Tissues were homogenized and extracted with ice‐cold methanol/chloroform/water (1:1:1) and vigorously vortexed. Samples were stored at 4°C overnight. After phase separation by centrifugation at 20,000 *g* at 4°C for 30 min, the polar water‐methanol phase containing water‐soluble cellular metabolites was retained. Methanol was removed by evaporation, and the samples were lyophilized. The organic phase (lipid phase) was collected, and chloroform was evaporated under nitrogen. Both phases were stored at −20°C. High‐resolution 1H NMR analyses were performed at 25°C at 400 MHz (9.4 T Bruker AVANCE spectrometer; Karlsruhe, Germany, Europe) using acquisition pulses, water pre‐saturation, data processing and peak area deconvolution as previously described (Metere et al., 2020). Quantification of individual metabolites was obtained from peak areas applying the correction factors determined by experiments at equilibrium of magnetization ([90]° pulses, 30.00‐s inter‐pulse delay). Metabolite quantification was expressed as nanomoles/mg tissue. All data were calculated as mean ± SD.

### 4.5 Principal component and correlation analyses

The metabolic profile of the animals was analysed by the PCA approach using single mouse as statistical units and metabolite concentrations as variables. PCA is the most popular multivariate analysis technique whose aim is the projection of an initially n‐dimensional space to a p < n space spanned by p mutually independent axes (components) preserving the relevant (signal) part of original information. The reduction in dimensionality is achieved by the computation of the eigenvectors correspondent to the higher eigenvalues of the among‐variables (here variables = metabolites) correlation matrix. This implies PCA solution keeps track of the actual among‐variables correlation present in the studied data set. Here, we compute two independent PCAs for fasting and non‐fasting animals in which the main order parameters (most important correlation fluxes) are different, being general metabolic “tone” for no fasting and amino acid metabolism for fasting. The components are extracted in order of percentage of variance explained, and their meaning is assigned by the component loading pattern being component loading the Pearson correlation coefficient between original variables and components. Each statistical unit (animals in this case) is assigned a score for each extracted component, and the scores are normalized so to have zero mean and unit standard deviation on the entire set.

### 4.6 Single‐cell gel electrophoresis analysis (comet assay)

The standard alkaline comet assay (pH > 13) was performed as described in Collins et al. (2008). Ten μl whole blood was mixed with 0.75% low‐melting‐point agarose (90 μl) and seeded on a microscope slide precoated with 1% normal melting‐point agarose. The slides were dipped in a lysis solution (2.5 M NaCl, 10 mM Tris–HCl, 100 mM EDTA, pH 10, with 1% Triton and 10% DMSO freshly added) for 1 day at 4°C and then subjected to electrophoresis for 20 min at 25 V and 300 mA at 4°C, preceded by a 15‐min incubation in electrophoresis buffer (1 mM EDTA, 300 mM NaOH, pH 13) to allow DNA unwinding. Slides were neutralized (0.4 M Tris–HCl, pH 7.5) and stained with ethidium bromide (20 μg/ml, 50 μl). Nucleoids were examined at 400× magnification with a fluorescence microscope (Axioskop 2, Zeiss) associated with a Comet Assay III programme. To evaluate DNA damage, computer‐generated %DNA in the tail (Tail Intensity, TI) values was used. Enzyme‐modified comet assay was carried out according to Collins et al. (2008). After lysis, 30‐min incubation at 37°C with Fpg or enzyme buffer was performed before 40‐min incubation in electrophoresis buffer and 30‐min electrophoresis at 25 V and 300 mA at 4°C.

To quantify the number of SSBs in the experimental conditions of our comet assays, a calibration curve was generated using lymphocytes of wild‐type mice exposed to ^137^Cs γ radiation in the dose range 1–8 Gy (dose rate 0.7 Gy/min). To transform percentage of tail DNA to the number of SSBs per genome, we used a value of 0.29 strand breaks/10^9^ Da per Gy for the average strand break yield and a diploid cell content of 4 × 10^12^ Da DNA.

### 4.7 Micronucleus assay in peripheral blood reticulocytes and CREST staining

Micronuclei were analysed by acridine orange fluorescent staining. Five microlitres of whole blood were distributed on slides coated with 10 μl of a solution of 1 mg/ml acridine orange (Sigma, Italy) in distilled water and examined under the fluorescence microscope. Slides were coded, and the frequency of micronucleated reticulocytes in 1000 peripheral blood reticulocytes was determined.

To characterize the content of micronuclei, immunostaining with CREST antibodies was performed. Slides were incubated for 10 min at room temperature (rt) in KCM buffer (120 mM KCl, 20 mM NaCl, 10 mM, Tris–HCl (pH 8), 0.5 M EDTA and 0.1% Triton X‐100), washed in 1x PBS for 3 min at r.t., incubated for 1 h at 37°C with 30 μl of anti‐kinetochore antibody solution (antibodies incorporated, Davies, CA) (1:1 in 1x PBS), gently washed in 4x SSC/0.05% Tween‐20 for 3 min and incubated with 50 μl of PNM buffer (0.1 M Na2HPO4, 0.1 M NaH2PO4, 0.1% NP‐40, pH 8.0 and 5% non‐fat dry milk) for 6 min at room temperature. Fifty μl of FITC goat anti‐human IgG (Chemicon International, CA) (1:60 in PNM) was added on each slide for 1 h at 37°C. Slides were counterstained with 10 μl of DAPI (0.5 μg/ml). One hundred micronuclei for each experimental point were analysed and classified as CREST‐negative (no signal) and CREST‐positive (signals).

### 4.8 Statistical analysis

PCA was adopted to analyse metabolomic data. The goodness of fit of linear relation was assessed by the Pearson correlation coefficient. Inferential statistics on both component scores and raw variables was assessed by means of analysis of variance and Student's *t* test. Data are expressed as mean ± SE for biological replicates with comparisons carried out using two‐tailed Student's *t* test. The limit for statistical significance was set at *p*‐values <0.05.

## CONFLICT OF INTEREST

The authors declare no competing interests.

## AUTHOR CONTRIBUTIONS

GDL, LC, MC, FDB, AM, FM, RB, MEP, BR and ES performed the experiments. MB, AG, EI, PK, FM, RM and SP analysed and interpreted data, and prepared the original draft. MB, PK, FM and RM reviewed and edited the paper.

## Supporting information

Table S1‐S6

## Data Availability

The data generated or analysed during this study and included in this published article (and its Supplementary Information files) will be available upon reasonable request to the corresponding author.
