# Supplementary material for: Young transgenic hMTH1 mice are protected against dietary fat‐induced metabolic stress—implications for enhanced longevity
Source: Aging Cell. 2022 Jun 6;21(7):e13605. doi: 10.1111/acel.13605 (PMC9282835; doi:10.1111/acel.13605)
Supplement: Supplementary file 1 — Table S1‐S6 [file ACEL-21-e13605-s001.docx]

**SUPPLEMENTARY DATA**

**Table 1S: Absolute quantification of aqueous liver metabolites in 2 month-old wild-type and hMTH1 mice maintained in SD and HFD^a^**

|  | **Diet** | **SD** | | **HFD^b^** | |
| --- | --- | --- | --- | --- | --- |
|  | **Genotype** | **WT** | **hMTH1** | **WT** | **hMTH1** |
| **Glucose**  **metabolism** | **D-glucose** | 19.79±5.01 | 21.30±2.84 | 29.55±4.91 | 36.80±9.26 |
|  | **Lactic acid** | 5.89±1.15 | 7.36 ±0.45 | 5.86±0.34 | 8.82±1.37 |
| **Nucleotide metabolism** | **AXP** | 3.41±0.64 | 4.61±0.59 | 4.47±0.58 | 5.39±0.23 |
|  | **NAD+NADP** | 0.75±0.10 | 0.63±0.10 | 0.76±0.14 | 0.59±0.07 |
|  | **UXP** | 2.49±0.38 | 2.81±0.36 | 2.56±0.42 | 3.64±0.63 |
|  | **UDP+UTP** | 1.16±0.17 | 1.19±0.16 | **0.66±0.17** | **1.31±0.20** |
| **One carbon metabolism** | **Formic acid** | 2.68±0.62 | 3.21±0.04 | 3.65±0.49 | 4.28±0.18 |
| **Amino acids metabolism** | **Lphenylalanine** | 0.27±0.04 | 0.22±0.0.04 | **0.06±0.03** | **0.24±0.02** |
|  | **L-tyrosine** | 0.83±0.12 | 0.76±0.15 | **0.27±0.16** | **0.91±0.11** |
|  | **L-histidine** | 0.84±0.12 | 0.72±0.13 | **0.36±0.16** | **0.90±0.10** |
|  | **L-glycine** | 2.94±0.66 | 4.12±0.47 | **2.94±0.55** | **5.36±0.54** |
|  | **L- glutamic acid** | 3.52±0.68 | 4.51±0.50 | 3.60±0.09 | 4.87±0.16 |
|  | **L- valine** | 0.89±0.10 | 1.50±0.26 | 1.10±0.17 | 1.91±0.32 |
|  | **L- glutamine** | 4.11±0.77 | 5.77±0.50 | 5.40±0.26 | 6.90±0.62 |
|  | **L _isoleucine** | 2.92±0.40 | 5.27±0.88 | **3.32±0.15** | **6.78±1.31** |
|  | **L-aspartic acid** | 0.70±0.13 | 0.84±0.13 | 0.81±0.03 | 0.93±0.07 |
|  | **L-alanine** | 5.30±0.68 | 7.09±0.65 | 4.90±1.24 | 6.82±0.55 |
| **Redox balance metabolism** | **Glutathione** | 8.41±1.86 | 8.91±0.99 | 8.79±0.31 | 11.60±2.21 |
|  | **taurine** | 1.69±0.28 | 2.10±0.31 | 2.47±0.16 | 3.14±0.41 |
| **Lipid metabolism** | **GPC** | 0.77±0.21 | 1.12±0.17 | 1.59±0.44 | 1.11±0.10 |
|  | **PCho** | 0.82±0.25 | 0.98±0.16 | 1.32±0.23 | 1.74±0.44 |
|  | **Cho** | 0.41±0.16 | 0.86±0.09 | 1.39±0.51 | 1.26±0.09 |
|  | **Etn** | 2.04±0.45 | 2.20±0.40 | 5.14±1.63 | 3.80±0.46 |
|  | **Myo-inosytol** | 9.56±1.69 | 11.34±1.46 | 11.05±1.09 | 14.45±1.51 |
| **Lipid and amino acid metabolism** | **Acetic acid** | 3.22±0.62 | 4.52±0.53 | 3.50±0.49 | 5.70±0.59 |
| **TCA cycle** | **Fumaric acid** | 0.28±0.04 | 0.29±0.03 | 0.15±0.08 | 0.29±0.04 |
|  | **Succinic acid** | 0.50±0.11 | 0.52±0.03 | 0.61±0.12 | 0.54±0.04 |
|  | **Total creatine** | 0.90±0.15 | 1.85±0.45 | 1.21±±0.07 | 1.42±0.02 |

**^a^** Data expressed as nmole/mg tissue ± SE

**^b^** The statistically significant differences between genotypes are in indicated in bold.

Abbreviations: AXP = (AMP+ADP+ATP); UXP= (UMP+UDP+UTP); SD (standard diet); HFD (high fat diet); GPC (glycerophopshocholine); pCho (phosphocholine); Cho (choline);Etn (ethanolamine)

**Table 2S: Absolute quantification of aqueous liver metabolites 7 month-old wild-type and hMTH1 mice maintained in in SD and HFD^a^**

|  | **Diet** | **SD^b^** | | **HFD** | |  |
| --- | --- | --- | --- | --- | --- | --- |
|  | **Genotype** | **WT** | **hMTH1** | **WT** | **hMTH1** |  |
| **Glucose**  **metabolism** | **D-glucose** | 13.76±1.52 | 7.94 ± 2.06 | 18.45 ±1.09 | 18.20±5.54 |  |
|  | **Lactic acid** | 4.32 ± 0.28 | 3.77 ± 0.36 | 4.44 ± 0.45 | 4.48 ± 0.42 |  |
| **Nucleotide metabolism** | **AXP** | **0.53 ± 0.08** | **0.99 ± 0.09** | 0.62 ± 0.09 | 0.77 ±0.17 |  |
|  | **NAD+NADP** | **0.13 ± 0.02** | **0.37 ± 0.03** | 0.16 ± 0.06 | 0.31 ± 0.01 |  |
|  | **UXP** | 0.63 ± 0.04 | 1.06 ± 0.11 | 0.75 ± 0.05 | 0.88 ± 0.17 |  |
|  | **UDP+UTP** | 1.80 ± 0.21 | 1.28 ± 0.28 | 2.04 ± 0.19 | 2.01 ± 0.25 |  |
| **One carbon metabolism** | **Formic acid** | 0.18 ± 0.05 | 0.21±0.06 | 0.21±0.05 | 0.57±0.33 |  |
| **Amino acids metabolism** | **Lphenylalanine** | 0.24 ± 0.07 | 0.25 ± 0.03 | 0.35 ± 0.07 | 0.33 ±0.03 |  |
|  | **L-tyrosine** | 0.36 ± 0.04 | 0.42 ± 0.09 | 0.42 ± 0.06 | 0.53 ± 0.06 |  |
|  | **L-histidine** | 0.92 ± 0.07 | 2.29 ± 0.54 | 0.93 ± 0.12 | 1.72 ± 0.33 |  |
|  | **L-glycine** | 2.01 ± 0.08 | 2.70 ± 0.53 | 1.90 ± 0.08 | 2.27 ± 0.23 |  |
|  | **L- glutamic acid** | **2.03 ± 0.12** | **4.42 ± 0.77** | 2.12± 0.15 | 2.61 ± 0.51 |  |
|  | **L- valine** | 0.56 ± 0.10 | 0.89 ± 0.15 | 0.59 ± 0.04 | 0.65 ± 0.13 |  |
|  | **L- glutamine** | 3.02 ± 0.21 | 1.96 ± 0.31 | 2.94 ± 0.23 | 2.77 ± 0.15 |  |
|  | **L _isoleucine** | 0.52 ± 0.07 | 0.98 ± 0.20 | 0.51 ± 0.05 | 0.67 ± 0.13 |  |
|  | **L-aspartic acid** | 0.85 ± 0.15 | 1.29 ± 0.13 | 0.73 ± 0.17 | 0.75 ±0.31 |  |
|  | **L-alanine** | 3.57 ± 0.23 | 4.19 ± 0.95 | 4.16 ± 0.70 | 3.85 ± 0.36 |  |
| **Redox balance metabolism** | **Glutathione** | **4.99 ± 0.40** | **3.27 ± 0.33** | 5.10 ± 0.21 | 4.62 ± 0.44 |  |
|  | **taurine** | 1.10 ± 0.15 | 1.11 ± 0.11 | 1.11 ± 0.13 | 0.90 ± 0.17 |  |
| **Lipid metabolism** | **GPC** | 0.31 ± 0.03 | 0.47 ± 0.12 | 0.42 ± 0.10 | 0.35 ± 0.07 |  |
|  | **PCho** | 0.83 ± 0.12 | 0.49 ± 0.17 | 0.92 ± 0.08 | 1.24± 0.15 |  |
|  | **Cho** | 0.36 ± 0.07 | 0.75 ± 0.22 | 0.33 ± 0.08 | 0.36 ± 0.08 |  |
|  | **Etn** | 0.22 ± 0.06 | 0.25 ± 0.04 | 0.15 ± 0.04 | 0.21 ± 0.05 |  |
|  | **Myo-inositol** |  |  |  |  |  |
| **Lipid and amino acid metabolism** | **Acetic acid** | 0.45 ± 0.03 | 0.62 ± 0.15 | 0.32 ± 0.02 | 0.46 ± 0.08 |  |
| **TCA cycle** | **Fumaric acid** | 0.12 ± 0.01 | 0.15 ± 0.05 | 0.13 ± 0.02 | 0.18 ± 0.01 |  |
|  | **Succinic acid** | 0.23 ± 0.03 | 0.27 ± 0.03 | 0.35 ± 0.06 | 0.29 ± 0.04 |  |
|  | **Total creatine** | 0.26 ± 0.03 | 0.42 ± 0.09 | 0.26 ± 0.03 | 0.27 ± 0.08 |  |

^a^ Data expressed as nmole/mg tissue ± ES.

**^b^** The statistically significant differences between genotypes are in indicated in bold.

Abbreviations: AXP = (AMP+ADP+ATP); UXP= (UMP+UDP+UTP); SD (standard diet); HFD (high fat diet); GPC (glycerophopshocholine); pCho (phosphocholine); Cho (choline);Etn (ethanolamine)

**Table 3S:** **PCA analysis performed on the entire set of metabolites analysed in 2 month-old wild-type and hMTH1 mice maintained in SD and HFD^a^**

| **Loading factor^a^** | | **PC1** | **PC2** | **PC3** | **PC4** | **PC5** |
| --- | --- | --- | --- | --- | --- | --- |
| **Proportion of total variance** | | 0.4718 | 0.1788 | 0.1031 | 0.0754 | 0.0473 |
| **Cumulative effect** | | 0.4718 | 0.6506 | 0.7536 | 0.8290 | 0.8763 |
|  | | | | | | |
| **Glucose**  **metabolism** | **D-glucose** | **0.65001** | 0.32296 | **0.54961** | -0.11312 | -0.27350 |
|  | **Lactic acid** | **0.91231** | -0.06199 | 0.22886 | -0.16672 | 0.01775 |
| **Nucleotide metabolism** | **AXP** | **0.91865** | 0.21910 | -0.04756 | 0.19241 | 0.03874 |
|  | **NAD-NADP** | 0.18683 | **-0.43509** | **0.44178** | **0.49963** | -0.12588 |
|  | **UXP** | **0.92737** | -0.09836 | 0.22218 | 0.14922 | -0.02765 |
|  | **UDP-UTP** | **0.73205** | **-0.65598** | 0.12146 | 0.00728 | 0.03804 |
| **One carbon metabolism** | **Formic acid** | **0.76898** | 0.35657 | 0.05787 | 0.27834 | -0.15298 |
| **Amino acids metabolism** | **Lphenylalanine** | 0.47449 | **-0.83935** | -0.03903 | -0.00192 | -0.02011 |
|  | **L-tyrosine** | **0.63773** | **-0.74453** | -0.08854 | -0.01394 | -0.02775 |
|  | **L-histidine** | **0.65308** | **-0.73485** | 0.03220 | 0.09032 | -0.01198 |
|  | **L-glycine** | 0.94278 | -0.14203 | 0.00063 | -0.00773 | -0.03501 |
|  | **L- glutamic acid** | **0.84080** | 0.18127 | -0.29123 | -0.09135 | 0.21930 |
|  | **L- valine** | **0.54431** | 0.10640 | -**0.70615** | -0.12044 | -0.33339 |
|  | **L- glutamine** | **0.86400** | 0.36373 | 0.03119 | -0.25747 | 0.16434 |
|  | **L _isoleucine** | **0.55204** | 0.06907 | -**0.75079** | -0.12659 | -0.25684 |
|  | **L-Aspartic acid** | **0.86059** | 0.18801 | -0.02176 | -0.03185 | 0.21773 |
|  | **L-alanine** | **0.58404** | -0.27182 | -0.25574 | -**0.56604** | 0.05609 |
| **Redox balance metabolism** | **Glutathione** | **0.87675** | 0.11081 | 0.35016 | -0.09856 | -0.14508 |
|  | **taurine** | **0.88934** | 0.30905 | 0.06171 | 0.07268 | -0.08875 |
| **Lipid metabolism** | **GPC** | **0.47243** | **0.48602** | -0.15503 | **0.55146** | 0.32371 |
|  | **PCho** | **0.63584** | 0.39104 | **0.45151** | -0.16225 | -0.16614 |
|  | **Cho** | 0.37875 | 0.71724 | -0.03548 | -0.15452 | -0.08120 |
|  | **Etn** | 0.28354 | **0.51238** | -0.16028 | **0.70338** | 0.11932 |
| **Lipid and amino acid metabolism** | **Acetic acid** | **0.79628** | 0.00895 | -**0.53031** | 0.05480 | 0.03344 |
| **TCA cycle** | **Fumaric acid** | **0.64236** | **-0.64969** | 0.10397 | 0.25094 | 0.20862 |
|  | **Succinic acid** | 0.24187 | 0.32058 | **0.51053** | -0.36872 | 0.00115 |
|  | **Total creatine** | 0.13215 | 0.07928 | 0.06821 | -0.32963 | 0.79155 |

^a^ Statistically significant values are shown in bold.

Abbreviations: AXP = (AMP+ADP+ATP); UXP= (UMP+UDP+UTP); SD (standard diet); HFD (high fat diet); GPC (glycerophopshocholine); pCho (phosphocholine); Cho (choline);Etn (ethanolamine)

**Table 4S:** **PCA analysis performed on the entire set of metabolites analysed in 7 month-old wild-type and hMTH1 mice maintained in SD and HFD^a^**

| **Loading factor^a^** | | **PC1** | **PC2** | **PC3** | **PC4** | **PC5** |
| --- | --- | --- | --- | --- | --- | --- |
| **Proportion of total variance** | | 0.3371 | 0.2676 | 0.1318 | 0.0686 | 0.0581 |
| **Cumulative effect** | | 0.3371 | 0.6046 | 0.7364 | 0.8051 | 0.8632 |
|  | | | | | | |
| **Glucose**  **metabolism** | **D-glucose** | -**0.4770** | **0.50741** | 0.26867 | **0.46279** | -0.27684 |
|  | **Lactic acid** | -0.2429 | **0.83683** | 0.10101 | 0.04581 | -0.19735 |
| **Nucleotide metabolism** | **AXP** | 0.31112 | -0.12544 | -**0.57178** | **0.72101** | -0.03303 |
|  | **NAD-NADP** | **0.58501** | **0.62055** | -0.33986 | -0.14326 | 0.21071 |
|  | **UXP** | 0.15939 | -0.11601 | -**0.75958** | **0.59673** | 0.03178 |
|  | **UDP-UTP** | -0.4361 | **0.78788** | -0.13444 | -0.16345 | 0.11962 |
| **One carbon metabolism** | **Formic acid** | 0.40519 | 0.18111 | **0.49220** | -0.17062 | 0.41749 |
| **Amino acids metabolism** | **Lphenylalanine** | -0.0204 | 0.37377 | 0.16501 | 0.12551 | **0.80023** |
|  | **L-tyrosine** | 0.12060 | **0.89057** | -0.29511 | 0.16713 | 0.14068 |
|  | **L-histidine** | **0.91521** | 0.33815 | -0.10165 | -0.00548 | 0.06090 |
|  | **L-glycine** | **0.81548** | 0.42843 | 0.03681 | -0.03135 | -0.33584 |
|  | **L- glutamic acid** | **0.97978** | 0.06595 | -0.04742 | 0.08852 | 0.00243 |
|  | **L- valine** | **0.90310** | 0.16485 | 0.13493 | -0.01723 | -0.16647 |
|  | **L- glutamine** | -0.41104 | 0.02549 | **0.75049** | 0.24136 | 0.04130 |
|  | **L _isoleucine** | **0.95553** | 0.18792 | 0.06035 | 0.05653 | -0.15170 |
|  | **L-Aspartic acid** | **0.72443** | -**0.50105** | 0.39217 | 0.16068 | 0.09432 |
|  | **L-alanine** | 0.46363 | **0.72966** | 0.13846 | 0.04977 | 0.05966 |
| **Redox balance metabolism** | **Glutathione** | -**0.55329** | 0.46581 | **0.54500** | 0.32506 | -0.08450 |
|  | **taurine** | 0.49458 | -0.16036 | **0.72290** | 0.16116 | -0.04055 |
| **Lipid metabolism** | **GPC** | 0.09347 | -**0.72874** | 0.05229 | 0.00403 | 0.08472 |
|  | **PCho** | -0.32911 | **0.61911** | 0.42685 | 0.36478 | -0.20520 |
|  | **Cho** | 0.31820 | **-0.76662** | -0.08337 | 0.37657 | 0.14309 |
|  | **Etn** | 0.32802 | -**0.59904** | 0.39569 | 0.25738 | 0.32349 |
| **Lipid and amino acid metabolism** | **Acetic acid** | **0.92334** | -0.00682 | -0.04568 | -0.17737 | -0.10120 |
| **TCA cycle** | **Fumaric acid** | **0.52742** | **0.81978** | -0.00569 | -0.03825 | 0.06261 |
|  | **Succinic acid** | -0.20949 | **0.58248** | -0.15201 | 0.17163 | 0.41078 |
|  | **Total creatine** | **0.91098** | 0.04257 | 0.34157 | 0.08464 | -0.09982 |

^a^ Statistically significant values are shown in bold.

Abbreviations: AXP = (AMP+ADP+ATP); UXP= (UMP+UDP+UTP); SD (standard diet); HFD (high fat diet); GPC (glycerophopshocholine); pCho (phosphocholine); Cho (choline);Etn (ethanolamine)

**Table 5S: Absolute quantification of aqueous liver metabolites in 2 month-old wild-type and hMTH1 mice kept in HFD followed by 20 hr fasting^a^**

|  | **Diet** | **SD** | | **HFD^b^** | |
| --- | --- | --- | --- | --- | --- |
|  | **Genotype** | **WT** | **hMTH1** | **WT** | **hMTH1** |
|  |  | | | | |
| **Glucose**  **metabolism** | **D-glucose** | 8.09±0.76 | 10.17±4.61 | 26.94±2.67 | 18.95±3.21 |
|  | **Lactic acid** | 5.35±0.18 | 5.84±0.96 | **6.51±0.04** | **5.55±0.30** |
| **Nucleotide metabolism** | **AXP** | 4.21±0.26 | 4.17±0.19 | 3.77±0.18 | 4.30±0.29 |
|  | **NAD+NADP** | 0.43±0.05 | 1.26±0.67 | 0.49±0.04 | 0.49±0.04 |
|  | **UXP** | 2.18±0.06 | 2.47±0.60 | 1.87±0.15 | 2.01±0.12 |
|  | **UDP+UTP** | 1.07±0.12 | 0.99±0.25 | 0.95±0.03 | 0.92±0.07 |
| **One carbon metabolism** | **Formic acid** | 3.15±0.55 | 2.59±0.20 | 2.31±0.21 | 2.94±0.32 |
| **Amino acids metabolism** | **Lphenylalanine** | **0.20±0.01** | **0.11±0.02** | 0.18±0.01 | 0.13±0.02 |
|  | **L-tyrosine** | **0.76±0.05** | **0.51±0.07** | 0.68±0.02 | 0.60±0.09 |
|  | **L-histidine** | **0.69±0.04** | **0.31±0.07** | **0.56±0.03** | **0.39±0.06** |
|  | **L-glycine** | 2.75±0.23 | 3.43±0.73 | 2.83±0.17 | 3.05±0.11 |
|  | **L- glutamic acid** | 4.07±0.30 | 4.66±0.33 | 4.22±0.53 | 5.13±0.41 |
|  | **L- valine** | 1.12±0.10 | 1.38±0.11 | 0.95±0.02 | 1.00±0.13 |
|  | **L- glutamine** | 4.75±0.35 | 5.40±0.31 | 4.86±0.34 | 5.46±0.31 |
|  | **L -isoleucine** | 4.56±0.21 | 5.07±0.48 | 3.25±0.03 | 3.76±0.45 |
|  | **L-aspartic acid** | **0.73±0.01** | **1.54±0.19** | 0.93±0.06 | 1.05±0.04 |
|  | **L-alanine** | 5.42±0.21 | 5.83±0.74 | 6.09±0.54 | 5.75±0.36 |
| **Redox balance metabolism** | **Glutathione** | **7.11±0.74** | **11.21±0.78** | 9.11±0.46 | 9.68±0.44 |
|  | **taurine** | 2.01±0.04 | 2.30±0.19 | 2.17±0.13 | 2.29±0.06 |
| **Lipid metabolism** | **GPC** | 1.13±0.27 | 0.70±0.03 | 0.59±0.10 | 0.37±0.04 |
|  | **PCho** | 0.46±0.24 | 0.93±0.13 | 1.10±0.13 | 1.09±0.09 |
|  | **Cho** | 0.80±0.12 | 1.42±0.25 | 0.83±0.11 | 1.05±0.10 |
|  | **Etn** | **2.18±0.18** | **4.19±0.37** | 3.30±0.48 | 3.61±0.21 |
|  | **Myo-inositol** | 8.02±0.59 | 8.31±0.15 | 9.24±0.21 | 9.96±0.46 |
| **Lipid and amino acid metabolism** | **Acetic acid** | 4.42±0.24 | 5.45±0.40 | 4.07±0.34 | 4.47±0.15 |
| **TCA cycle** | **Fumaric acid** | 4.60±0.32 | 5.70±0.94 | 4.23±0.31 | 4.46±0.20 |
|  | **Succinic acid** | 0.59±0.10 | 0.71±0.07 | 0.72±0.05 | 0.85±0.10 |
|  | **Total creatine** | 1.42±0.14 | 1.60±0.09 | **1.06±0.05** | **1.34±0.07** |

^a^ Data expressed as nmole/mg tissue ± SE.

^b^ The statistically significant differences between genotypes are in indicated in bold.

Abbreviations: AXP = (AMP+ADP+ATP); UXP= (UMP+UDP+UTP); SD (standard diet); HFD (high fat diet); GPC (glycerophopshocholine); pCho (phosphocholine); Cho (choline);Etn (ethanolamine)

**Table 6S:** **PCA analysis performed on metabolites of 2 month mice kept in HFD followed** **by 20 hr fasting**

| **Loading factor^a^** | | **Factor1** | **Factor2** | **Factor3** | **Factor4** | **Factor5** |
| --- | --- | --- | --- | --- | --- | --- |
| **Proportion of total variance** | | 0.3985 | 0.1582 | 0.0954 | 0.0822 | 0.0619 |
| **Cumulative effect** | | 0.3985 | 0.5567 | 0.6521 | 0.7343 | 0.7962 |
|  | | | | | | |
| **Glucose**  **metabolism** | **D-glucose** | **0.60220** | -0.01477 | -**0.66032** | 0.12892 | -0.16460 |
|  | **Lactic acid** | **0.87586** | -0.16753 | -0.21737 | 0.14007 | -0.09682 |
| **Nucleotide metabolism** | **AXP** | **0.89678** | 0.16392 | -0.00924 | -0.11820 | 0.11888 |
|  | **NAD-NADP** | 0.15322 | -0.02195 | -0.01641 | 0.32512 | **0.74831** |
|  | **UXP** | **0.86575** | -0.21630 | -0.21518 | 0.06779 | 0.30557 |
|  | **UDP-UTP** | **0.65030** | -0.57083 | 0.12768 | 0.35828 | 0.22919 |
| **One carbon metabolism** | **Formic acid** | **0.73023** | 0.10748 | -0.24107 | -0.27812 | 0.17735 |
| **Amino acids metabolism** | **Lphenylalanine** | 0.42440 | **-0.80160** | 0.22379 | 0.16697 | -0.03803 |
|  | **L-tyrosine** | **0.55078** | **-0.66545** | 0.33432 | 0.21538 | -0.07449 |
|  | **L-histidine** | **0.56907** | **-0.77789** | 0.05483 | 0.05892 | 0.02820 |
|  | **L-glycine** | **0.91882** | -0.16748 | -0.02351 | 0.02192 | 0.05457 |
|  | **L- glutamic acid** | **0.63365** | 0.41817 | 0.38294 | 0.09080 | -0.18837 |
|  | **L- valine** | **0.60388** | 0.00689 | 0.45603 | -0.46253 | -0.04268 |
|  | **L- glutamine** | **0.85665** | 0.33810 | -0.04707 | -0.00086 | -0.25300 |
|  | **L _isoleucine** | **0.58180** | 0.01543 | **0.56073** | -0.43489 | -0.02715 |
|  | **L-Aspartic acid** | 0.45768 | **0.51861** | 0.29605 | 0.45050 | 0.32391 |
|  | **L-alanine** | **0.54033** | -0.18036 | 0.35974 | 0.10880 | -**0.51744** |
| **Redox balance metabolism** | **Glutathione** | **0.79184** | 0.29112 | -0.10708 | 0.36911 | -0.08235 |
|  | **taurine** | **0.86140** | 0.30499 | -0.07125 | 0.03167 | 0.00010 |
| **Lipid metabolism** | **GPC** | 0.46115 | 0.02926 | -0.14660 | -**0.58721** | 0.30994 |
|  | **PCho** | **0.62315** | 0.19778 | -**0.52086** | 0.18498 | -0.20132 |
|  | **Cho** | 0.36642 | **0.71720** | 0.01191 | -0.17218 | -0.14329 |
|  | **Etn** | 0.26764 | **0.60371** | -0.01542 | -0.25534 | 0.36563 |
| **Lipid and amino acid metabolism** | **Acetic acid** | **0.69265** | 0.22103 | **0.61251** | -0.12881 | 0.05056 |
| **TCA cycle** | **Fumaric acid** | -0.2626 | 0.44716 | 0.42296 | **0.55838** | 0.23859 |
|  | **Succinic acid** | -0.0188 | **0.56649** | 0.04999 | 0.53781 | -0.26492 |
|  | **Total creatine** | 0.15203 | 0.11674 | 0.06139 | 0.00353 | -0.17600 |

^a^ Statistically significant values are shown in bold.

Abbreviations: AXP = (AMP+ADP+ATP); UXP= (UMP+UDP+UTP); SD (standard diet); HFD (high fat diet); GPC (glycerophopshocholine); pCho (phosphocholine); Cho (choline);Etn (ethanolamine)
